# Thrombolysis Enhancing by Magnetic Manipulation of Fe_3_O_4_ Nanoparticles

**DOI:** 10.3390/ma11112313

**Published:** 2018-11-17

**Authors:** Qian Li, Xiaojun Liu, Ming Chang, Zhen Lu

**Affiliations:** 1 The State Key Laboratory of Digital Manufacturing Equipment and Technology, Huazhong University of Science and Technology, Wuhan 430074, China; LQ9365@sina.com (Q.L.); mexjl@163.com (X.L.); 2 Department of Mechanical Engineering, Chung Yuan Christian University, Chung Li 32023, Taiwan; 3 College of Mechanical Engineering and Automation, Huaqiao University, Xiamen 361021, Fujian, China; 4 School of Economics and Management, Shanghai University of Electric Power, Shanghai 200090, China; luzhen89@163.com

**Keywords:** thrombolysis enhancing, Fe_3_O_4_ nanoparticles, rotating magnetic field, convective diffusion, low-dose urokinase

## Abstract

In this paper, an effective method of accelerating urokinase-administrated thrombolysis through a rotating magnetic field (RMF) of guided magnetic nanoparticles (NPs) in the presence of low-dose urokinase is proposed. The dispersed Fe_3_O_4_ NPs mixed with urokinase were injected into microfluidic channels occluded by thrombus prepared in vitro. These magnetic NPs aggregated into elongated clusters under a static magnetic field, and were then driven by the RMF. The rotation of Fe_3_O_4_ aggregates produced a vortex to enhance the diffusion of urokinase to the surface of the thrombus and accelerate its dissolution. A theoretical model based on convective diffusion was constructed to describe the thrombolysis mechanism. The thrombus lysis speed was determined according to the change of the thrombus dissolution length with time in the microfluidic channel. The experimental results showed that the thrombolysis speed with rotating magnetic NPs is significantly increased by nearly two times compared with using the same dose of pure urokinase. This means that the magnetically-controlled NPs approach provides a feasible way to achieve a high thrombolytic rate with low-dose urokinase in use.

## 1. Introduction

Thromboembolism is a common cardiovascular disease that threatens the life of human beings [1,2]. After a thrombus forms and impedes a vessel, the oxygen and nutrition in the blood circulation system cannot be transported to the normal tissues and cells; then, a serious of complications may follow [3,4]. Many clinical trials have proved the value of thrombolytic agents, such as urokinase [5], streptokinase [6,7], tissue-type plasminogen activator (t-PA) [8], and so on in the treatment of venous and arterial thromboembolic diseases. Large therapeutic doses of the agents are always needed to lyse the clot and reestablish antegrade blood flow for a shortened thrombolysis time [9,10]. However, this may in turn result in the potential risks of symptomatic intracranial hemorrhages [11,12,13]. Therefore, other security methods to control the dose while improving the thrombolytic therapy rate should be sought. A few promising methods have been put forward. As a non-invasive technique, ultrasound is utilized to reduce the infusion time and provide a great incidence of complete lysis with a low rate of bleeding in treatment of deep-vein thrombosis (DVT) [14,15]. The ultrasound accelerates thrombolysis by disaggregating the fibrin matrix and exposing additional plasminogen receptor sites to the thrombolytic agents [16]. An approximately half-dose reduction in urokinase, t-PA, and recombinant tissue plasminogen activator (r-tPA) is found for thrombolysis by using ultrasound. However, several safety factors, such as vascular wall injury and subsequent inflammation, still need to be considered to achieve an optimal risk–benefit ratio [17]. Another typical interventional effective therapy is nanomedicine delivery, which results from the combination of nanotechnology and biomedicine [18,19]. Coated onto the surface [20,21] or encapsulated into polymeric shells [22], the magnetic nanoparticles (NPs) can transport the pharmaceuticals’ molecules to the target lesion under the control of an external magnetic field. The conjugation of urokinase and NPs reduces the free diffusion of drug molecules in the flow, and raises the amount of medicament to thrombi. As a result, the efficiency in thrombolysis can be improved compared with pure urokinase application, which has been proven in our previous studies [23]. Shear-activated nanotherapeutics for drug targeting is another controlling method of NPs [24,25]. The drug-coated NP aggregates fall apart and release the drugs only when encountering high fluid shear stress, which is the result of fluid flow at the narrow gap between the thrombus and the vessel wall. It helps restore the fluid flow at lower dose of drugs than free ones. However, the common process for the connection of drugs and NPs is always complicated, and may contain chemicals that are not suitable for use in vivo [26,27].

In order to solve the above problems, a new idea for the further diffusion of drug molecules toward the surface of thrombus occlusion by manipulating the movement of magnetic NPs is proposed to improve the thrombolysis efficiency. Our other study showed that Fe_3_O_4_ NPs could aggregate into microrods under a static magnetic field, and then be controlled to move and rotate, thus producing a flow vortex and breaking thrombus directly [28]. However, small thrombus fragments may be generated during the crushing process, which may lead to new occlusion in some smaller capillaries [29,30]. Here, the diffusion of thrombotic urokinase is enhanced under the influence of the convection flow, and then, more drugs can act on the thrombi to accelerate thrombolytic therapy than a pure agent without NPs. Since the thrombus is dissolved by drugs instead of being ablated by direct impact with the vortex flow or even the aggregates, no small blood clots will be produced. A thrombolysis model based on convection-enhanced diffusion theory is constructed to explain the thrombolysis acceleration process. The results of the thrombolytic experiments show that it is possible to apply this method in clinical practice in the future.

## 2. Materials and Methods

### 2.1. Preparation and Characterization of Magnetic Fe_3_O_4_ NPs

In this study, the magnetic Fe_3_O_4_ NPs were prepared with a modified co-precipitation method [21]. First, 1.0 g of FeCl_2_·4H_2_O and 2.7 g of FeCl_2_·6H_2_O were mixed in 50 mL of deionized water and heated to 80 °C under the protection of nitrogen atmosphere. Then, 25 mL of ammonia solution was added into the hot mixture drop by drop. The mixture was stirred and heated at 80 °C for 60 min. Subsequently, 2 g of citric acid dissolved in 10 mL of deionized water was added, and the temperature was raised to 90 °C under continuous stirring for 90 min. After cooling to room temperature, the black products were absorbed to the bottom of the reaction beaker by a strong magnet, and then the supernatant was removed. Subsequently, the sediment was washed four to five times until a pH of 7~8 was reached. Finally, the sediment was dried under flowing nitrogen at room temperature for one hour to obtain citric acid-coated Fe_3_O_4_ NPs.

**Figure 1:**
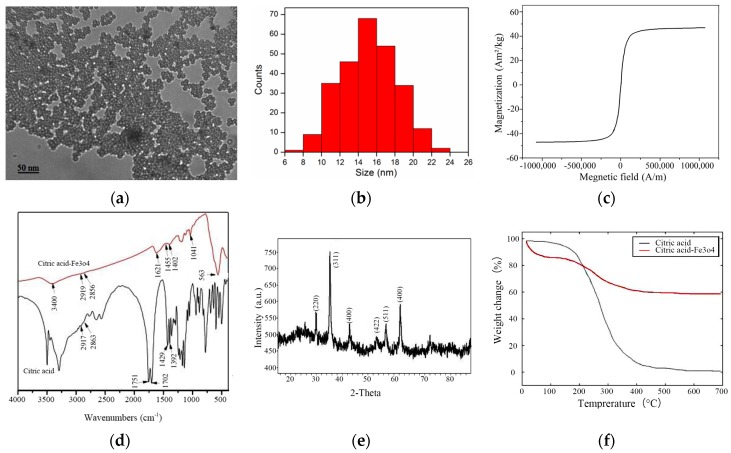
Characterization of prepared Fe_3_O_4_ nanoparticles (NPs): (**a**) TEM micrograph of NPs; (**b**) Size distribution of NPs measured by dynamic light scattering; (**c**) Field dependence of magnetization (Magnetization vs. Magnetic field) plot of NPs at 25 °C; (**d**) Fourier transform infrared (FTIR) spectra of pure citric acid and citric acid-coated NPs; (**e**) X-ray diffraction (XRD) pattern of prepared NPs; (**f**) Thermogravimetric (TG)-DTA plots of citric acid and citric acid-coated NPs.

The morphology of the synthesized NPs was observed by transmission electron microscopy (TEM) (JEM-2010, JOEL, Tokyo, Japan). Figure 1a shows the TEM micrograph of prepared citric acid-coated NPs. The colloidal stability of NPs was further investigated with a dynamic light scattering particle size distribution analyzer (LB-550, Horiba, Japan). Figure 1b shows that the NPs are uniformly dispersed with an average diameter of 15 nm. The magnetization of NPs, which was determined by a vibrating sample magnetometer (JDM-13, Jilin University, Jilin, China), was evaluated by sweeping the applied field from −795 kA/m to 795 kA/m at room temperature. Figure 1c shows that no hysteresis was detected, and the magnetization saturates at 47.6 Am^2^/kg. The superparamagnetic behavior is indicated by zero coercivity and zero remanence on the magnetization curve. The infrared absorption investigations of NPs were carried out with a Fourier transform infrared absorption (FTIR) (VERTEX 70, Bruker, Germany). Figure 1d shows the FTIR spectra of the pure citric acid and citric acid-coated NPs. Broad vibration bands are found for citric acid, but those of citric acid-coated NPs are few. The 1702 cm^−1^ peak assignable to the C=O vibration from the COOH group of citric acid shifts to 1621 cm^−1^ at citric acid-coated NPs, which reveals that the acid is bound onto the surface of the NPs by chemisorption of carboxylate ions. The bands at 1402 cm^−1^ and 1041 cm^−1^ in citric acid-coated NPs correspond to the symmetric stretching of the COO^−^ and OH groups of citric acid. The band at 563 cm^−1^ is the Fe–O stretching vibrational mode of Fe_3_O_4_. The structure of the NPs was studied by X-ray diffraction (XRD) (X’Pert PRO, PANalytical B.V., Almelo, Nederland) with diffraction angles from 10° to 90°. Figure 1e shows the XRD patterns for the samples. The patterns indicate a crystallized structure with peaks at 2θ = 30.1°, 35.4°, 43.1°, 53.4°, 57°, and 62.6°, which are assigned to (220), (311), (400), (422), (511), and (440) crystallographic faces of magnetite. The average size of the NPs can be evaluated from the (311) characteristic reflection as 14.2 nm, according to Scherrer’s formula. The thermogravimetric (TG) analysis of the NPs was done with a differential thermogravimetric analyzer (Diamond TG/DTA, PerkinElmer Instruments, Waltham, MA, USA). Figure 1f shows the TG-DTA plots of citric acid and citric acid-coated NPs. A sharp weight loss of citric acid-coated NPs of about 15% at 100 °C can be ascribed to the removal of water on the surface of citric acid-coated NPs. A weight loss of about another 25% at 400 °C can be attributed to the removal of citric acid on the surface of NPs.

### 2.2. Experimental System

**Figure 2:**
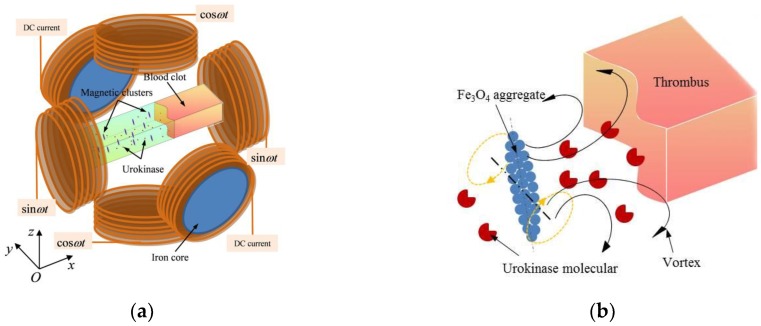
(**a**) Schematic of the magnetic control system; (**b**) The diffusion of urokinase is manipulated by the vortex induced by the rotation of rotating magnetic field (RMF)-guided aggregates.

Figure 2a shows the schematic diagram of the magnetic control system. A straight microfluidic channel made of polydimethylsiloxane (PDMS) with the lithography method was used to model the capillary vessel. A thrombus clot prepared in vitro is the same as those used by Serša et al. [31], which was injected into the channel with a transferpettor. The magnetic NPs’ fluids were injected into the channel gradually with a microfluidic pump. Three pairs of electromagnetic coils were adopted to produce a controlling magnetic field in the channel. Each coil was manually wound 400 turns, and the induced magnetic field was detected by a magnetic induction intensity meter (WT10E, Beijing Electronic Technology Co., Ltd., Beijing, China) with a resolution of 10 A/m and a range of 0 A/m to 4774 A/m. The static field related to the inputting direct current (DC) voltage and alternating field related to the alternating current (AC) voltage into the coils were detected and calibrated for controlling the magnetic field by changing the voltage directly. Upon manipulation, the electromagnetic coils in the *y* axis, fed with DC current, were used to magnetize and accumulate the NPs and help form aggregates, while the coils in the *x* and *z* axis, which were fed with an AC current, were used to produce a rotating magnetic field (RMF) for the rotation and translation of the NPs. As shown in Figure 2b, urokinase molecules were rapidly diffused to the surface of the thrombus through the vortex induced by the rotation of aggregates guided by the RMF to accelerate the thrombolysis.

**Figure 3:**
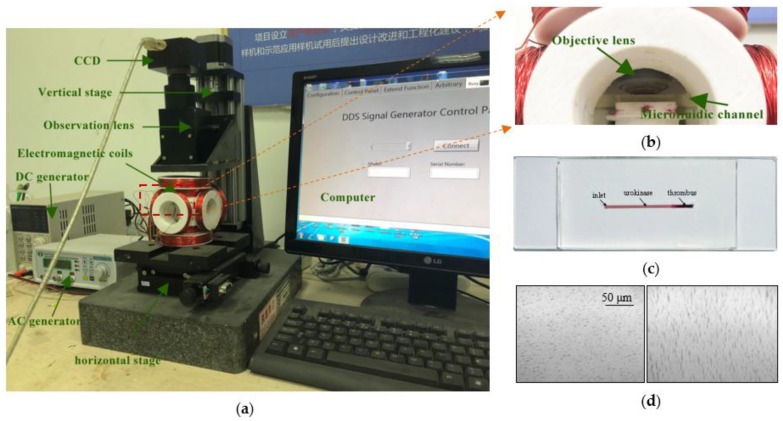
(**a**) Magnetic control and optical inspection system; (**b**) Microfluidic channel imaging setup; (**c**) Picture of the prepared thrombus-occluded channel; (**d**) Two images of NP aggregates in the microfluidic channel.

The movement of magnetic aggregates and the thrombolytic process were observed with an optical microscope system, as shown in Figure 3a. The electromagnetic coils were placed on a horizontal two-dimensional stage for adjusting the observation field. The microfluidic channel was placed on a plastic support plate to ensure that the thrombus sample was within the focal length of the objective lens, as shown in Figure 3b. Figure 3c is the picture of the prepared thrombus occlusion channel with a width of 0.8 mm, a depth of 0.2 mm, and a length of 25 mm. Figure 3d shows the NP aggregates formed in the microfluidic channel when current was introduced into coils from the DC and AC generators.

### 2.3. Thrombolysis Model in Microfluidic Channel

In principle, the lysis of the thrombus is a process of diffusion and chemical reaction. A model of urokinase-mediated thrombolysis based on convective diffusion theory is here established to evaluate the thrombolytic effect of rotating NP aggregates. Thrombolysis involves a series of enzyme-catalyzed reactions, which are mainly divided into three processes: (1) urokinase molecules diffuse to the surface of the thrombus and react with the plasminogen bonded on the fibrin; (2) plasminogen is activated and converted into enzyme plasmin, which is responsible for the degradation of fibrin into a fibrin-hydrolyzed product; (3) hydrolyzed products are released and dissolved to expose new plasminogen for reaction. Considering a diffusion controlled process, the reaction rate can be expressed as $r=4\pi Na(D_{UK}+D_{plm})C_{UK}C_{plm}$ [32], where *N* is Avogadro’s number, a is the encounter radius, $D_{UK}$ and $C_{UK}$ are the diffusion coefficient and concentration of urokinase, and $D_{plm}$ and $C_{plm}$ are the diffusion coefficient and concentration of plasminogen, respectively. Due to the plasminogen being bound on the fibrin, $D_{plm}$ can be seen as zero, and $C_{plm}$ can be seen as a constant, so *r* is mainly influenced by $D_{UK}$ and $C_{UK}$. Since the reaction obviously starts from the contact boundary between the thrombus and urokinase, the lysis speed of the thrombus $v_{lysis}$ can be defined here as the moving distance of the thrombus boundary per unit of time in the occluded microfluidic channel. Note that *r* reflects the reduction rate of the thrombus mass over time, and the cross-sectional area in the microchannel is constant, so $v_{lysis}$ is proportional to *r*. Therefore, the lysis speed can be expressed as $v_{lysis}=kC_{UK}D_{UK}$, where *k* is the reaction factor related to $v_{lysis}$ and $D_{UK}$$C_{UK}$, and is affected by *N*, a, and $C_{plm}$.

For the case of pure urokinase, the diffusion coefficient of urokinase in the channel is usually a constant. Once a vortex is caused by the rotation of RMF-guided aggregates, the previously diffused urokinase would rotate about the rotation axis of the aggregates. Thus, the diffusion of urokinase can be expressed as $D_{UK}=D_{f}+\Delta D_{UK}$, where $D_{f}$ is the self-diffusion factor induced by pure urokinase, $\Delta D_{UK}=bPe^{2}D_{f}$ [32] is the additional rotating diffusion factor, *b* is a proportional constant, and *Pe* is Peclet number. The Peclet number characterizes the ratio of the advection rate of a physical quantity by the flow to the diffusion rate of the same quantity driven by an appropriate gradient. In order to consider the convection created by the rotation of the paramagnetic particle chains, Biswal and Gast proposed the Peclet number as $Pe=\gamma /(D_{f}/l^{2})$ [33], where *γ* measures the time scale for stretching the fluid, and is proportional to the rotation angular velocity ω of the aggregate; l is the aggregate length; and $l^{2}/D_{f}$ represents the diffusion time over a chain length. The diffusion coefficient is thus given as:

(1) $$D_{UK}=D_{f}(1+b\omega^{2}l^{4}/D_{f}{}^{2})$$

As it was known, the length of aggregate *l* has a nonlinear relationship with magnetic field strength H and NPs concentration $C_{NP}$ [28,34], so the length can be expressed as $l^{4}=k\prime H^{\alpha }C_{NP}{}^{\beta }$, where α and β are polynomial orders, and k′ is a correction factor. Accordingly, the thrombus boundary moving speed can be obtained as:

(2) $$v_{lysis}=v_{lysis\_f}+\Delta v_{lysis}=v_{lysis\_f}(1+bk\prime \omega^{2}H^{\alpha }C_{NP}{}^{\beta }/D_{f}{}^{2})$$

where $v_{lysis\_f}=kC_{UK}D_{f}$ is the lysis speed induced by the self-diffusion of urokinase, and $\Delta v_{lysis}$ is the additional lysis speed caused by convective diffusion provided by the rotation of NPs.

## 3. Results and Discussions

### 3.1. Observation of Thrombolysis Enhancing by Magnetic Fe_3_O_4_ NPs

**Figure 4:**
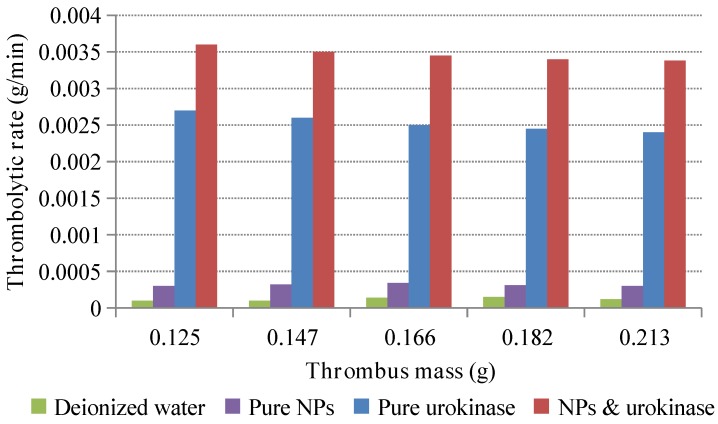
Comparison of thrombolytic rates for urokinase solutions with and without magnetic Fe_3_O_4_ NPs.

Observation of the thrombolysis effect for urokinase solutions with and without magnetic Fe_3_O_4_ NPs was first carried out. Four sets of small glass beakers were prepared, with each bottle containing 10 mL of deionized water and a little thrombus. The blood sample was collected from a healthy white mouse kindly provided by Tongji Hospital, Wuhan, China. The tiny amount of thrombus in each bottle was weighed with a precision balance in advance. Subsequently, 5 mg of urokinase was added to the first set of bottles, 5 mg of Fe_3_O_4_ NPs were added to the second set of bottles, a mixture of 5 mg of urokinase and 5 mg of Fe_3_O_4_ NPs was added to the third set of bottles, and the last set of bottles remained deionized water. The bottles with NPs were placed in the magnetic field area of our magnetic device. Each thrombolysis experiment was repeated five times, and the time from drug addition to the approximate disappearance of thrombus was recorded. The remaining thrombus in each bottle was removed from the bottle and weighed again at 30 min after each experiment. Figure 4 shows the experimental results, wherein the thrombolytic rate was calculated according to the amount of thrombus dissolution in the bottle over time. As shown, only a small amount of thrombolysis was observed in the control groups of pure water and NPs alone, which ensured that the thrombolytic rate was mainly based on the urokinase activity. The average thrombotic rate of pure urokinase is about 0.0025 g/min, which increased to 0.0035 g/min after adding the NPs. This shows that the thrombolytic effect is enhanced by 40% with the magnetic control of Fe_3_O_4_ NPs.

### 3.2. Thrombolysis Test In Vitro

**Figure 5:**
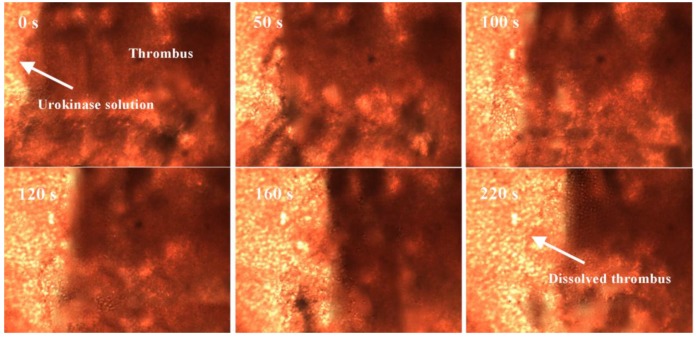
Image sequences demonstrating a pure urokinase-mediated thrombus removal process.

In order to further demonstrate the thrombolytic function of the rotating nanoparticle clusters in vitro, a comparison of thrombolysis effects in the microfluidic channel that is shown in Figure 3c was performed. First, the urokinase solution with a concentration of 50 μg/mL was inserted into the thromboembolic channel from the inlet. The thrombolytic process of pure urokinase was observed for 220 s with the microscopic system at a magnification of 50×. Figure 5 shows some sequence images of the thrombus removal process intercepted from a recording video. As shown in these images, the moving boundary of the thrombus indicates that the thrombus was gradually removed with the diffusion of the injection urokinase. In this case, the boundary moves at an average speed of about 20 μm/min.

**Figure 6:**
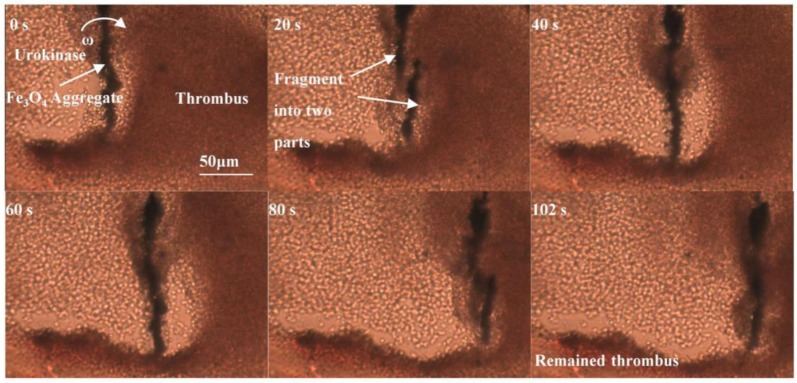
Image sequences of thrombus removal by the RMF-guided Fe_3_O_4_ NPs.

The in vitro thrombolysis experiment with Fe_3_O_4_ NPs and urokinase mixed solution in the microfludic channel was then studied. Figure 6 is the thrombus removal process in the channel using urokinase at a concentration of 50 μg/mL and NPs at a concentration of 10 mg/mL in an interval of 102 s. As shown in the image at 0 s, the NPs were first clustered into a microrod with a length of about 150 μm due to the static magnetic field, and then produced a rotational motion with an angular velocity of ω induced by the action of the RMF. The rotation of the Fe_3_O_4_ aggregate generated a vortex to enhance the diffusion of urokinase to the surface of the thrombus and accelerate its dissolution. Due to the cohesive force of the aggregate not being strong enough, sometimes, the aggregate fragmented into two parts, as shown in the images at 20 s and 80 s. However, the phenomenon of enhanced diffusion of urokinase was still continuing, and the thrombus of about 2 mg was ablated in about 102 s. Here, the thrombus lysis speed is roughly 36 μm/min. This indicates that the thrombolysis speed with the addition of rotating magnetic NPs is increased by about 1.8 times compared with using the same dose of pure urokinase.

**Figure 7:**
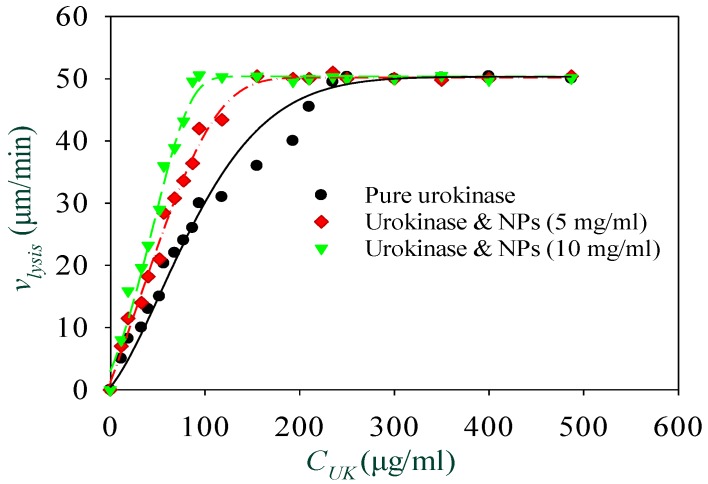
The thrombus lysis speed varies with the concentration of urokinase.

The thrombolysis experiments were repeated with different urokinase concentrations ranging from 0 to 500 μg/mL, and NPs concentrations at 0, 5 mg/mL, and 10 mg/mL. As shown in Figure 7, when the pure urokinase solution with concentration $C_{UK}$ varies from 0 to 250 μg/mL, the thrombolysis speed $v_{lysis}$ increases from 0 to 50 μm/min. While for $C_{UK}$ ≥ 250 μg/mL, the thrombus lysis speed no longer increases, but is approximately constant, which indicates that the urokinase in the microfluidic channel is almost saturated at the concentration of 250 μg/mL. For the solutions with NP concentrations of 5 mg/mL and 10 mg/mL, this happened at urokinase concentrations of 155 μg/mL and 94 μg/mL, respectively. This indicates that the magnetically-controlled NPs can significantly improve the thrombolysis effect when using low-dose urokinase.

**Figure 8:**
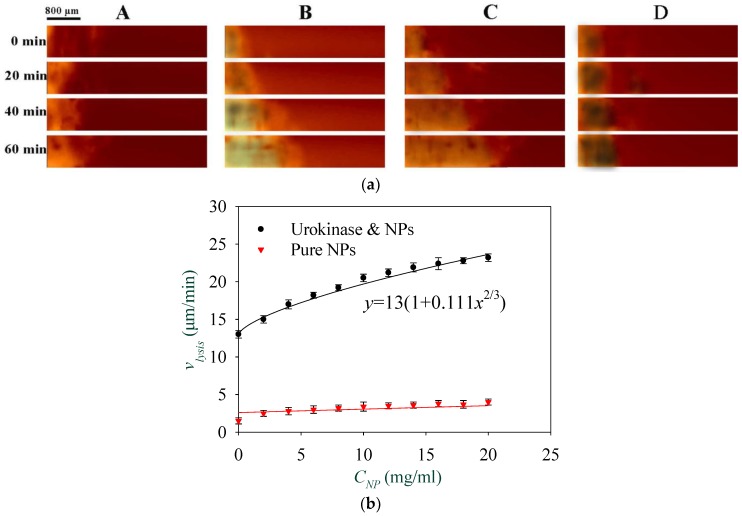
(**a**) Comparison of thrombolytic effects in four microfluidic channels with a urokinase concentration of 30 μg/mL and NPs at concentrations of 0 mg/mL, 5 mg/mL, and 10 mg/mL, and pure NPs (concentration of 5 mg/mL) without urokinase in channels *A*, *B*, *C*, and *D*, respectively; (**b**) Relationship between the thrombus lysis speed and the nanoparticle concentration with and without urokinase (concentration of 30 μg/mL).

The following experiment was conducted in order to investigate the effect of the concentration of NPs on thrombolysis speed. The solutions with a urokinase concentration of 30 μg/mL and NP concentrations from 0 to 20 mg/mL were injected into the microfluidic channels from the channel inlet at a constant flow rate of 5 μL/min. Figure 8a shows a comparison of four typical thrombolysis processes. The NPs concentration in channels *A*, *B*, and *C* were 0 mg/mL, 5 mg/mL, and 10 mg/mL, respectively. As a control, the thrombolysis effect for pure NPs solutions without urokinase was also observed. The images in channel *D* show the thrombolytic process of pure NPs with a concentration of 5 mg/mL. The magnetic field combined from a static magnetic field of 3000 A/m and an RMF of 3000 A/m with a driving frequency of 30 Hz was applied to the fluidic channel. The recording image at 0 min refers to 5 min after the injection of solution. From these images, the moving boundary of the thrombus shown in channels *B* and *C* with NPs and urokinase are obviously much faster than in channels *A* and *D*. These experiments were repeated three times, and an equation $v_{lysis}=13(1+0.111C_{NP}{}^{2/3})$ was obtained for solutions with a urokinase concentration of 30 μg/mL and shown in Figure 8b to describe the relationship between the boundary moving speed of the thrombus and the concentration of NPs. This indicates that the thrombolysis speed has a nonlinear relationship with the concentration of NPs, which is consistent with Equation (2), in which β=2/3. Here, the coefficient of determination $R^{2}$ was used to realize the accuracy of curve fitting. By varying β from 0.6 to 0.7 every 0.02, $R^{2}$ increased from 0.85 to 0.97, then decreased to 0.88, and the maximum value appeared at 0.66. This shows that there is still a slight deviation between the fitting curve and the experimental results, especially when $C_{NP}$ is between 10 and 15 mg/mL. As for the control with pure NPs, the thrombus lysis speed is only about three μm/min, even when the NPs concentration is as high as 20 mg/mL. The results show that the thrombolysis mainly relied on the urokinase activity, and the thrombolysis speed can be increased to nearly two times by adding NPs; thus, it is very feasible to enhance the thrombolysis efficiency by the magnetic manipulation of Fe_3_O_4_ NPs.

**Figure 9:**
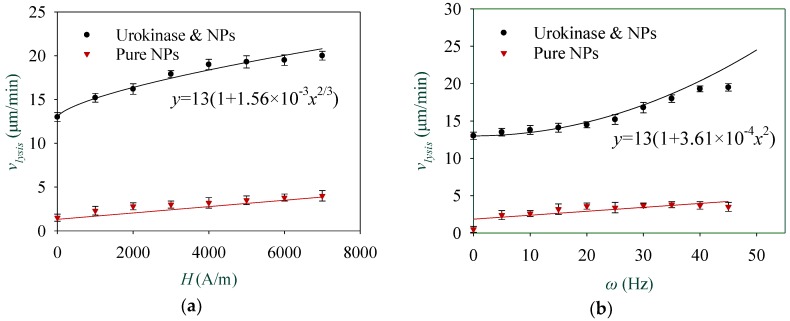
(**a**) Thrombolysis speed reveals a nonlinear dependence to the strength of the RMF; (**b**) Thrombolysis speed shows a quadratic dependence to the driving frequency of the RMF.

Finally, the effect of the magnetic field on thrombolysis was studied. By keeping the concentration of NPs at 5 mg/mL and urokinase at 30 μg/mL, the thrombus boundary moving images were recorded as the strength of the RMF was changed from 0 to 6000 A/m. As shown in Figure 9a, the relationship between the boundary moving speed of the thrombus and the strength of the RMF can be seen as $v_{lysis}=13(1+1.56\times 10^{-3}H^{2/3})$. This nonlinear correspondence is also consistent with Equation (2), in which α=2/3. Likewise, by varying α from 0.6 to 0.7 every 0.02, $R^{2}$ increased from 0.85 to 0.97; then, it decreased to 0.6, and reached its maximum at 0.66. Experiments concerning the relationship between the thrombolysis speed and the driving frequency of the RMF under static magnetic field of 3000 A/m and RMF of 3000 A/m were also carried out. As shown in Figure 9b, a quadratic dependence of $v_{lysis}=13(1+3.61\times 10^{-4}\omega^{2})$ was obtained. This is still consistent with Equation (2). For the frequency curve before 45 Hz, the coefficient of determination $R^{2}$ is 0.98, which means a good fitting. However, the thrombolysis speed is no longer promoted as the driving frequency reaches 40 Hz. This may be attributed to the cracking of most aggregates due to the increasing hydrodynamic force when the critical frequency of aggregates is reached [26]. In addition, experiments with a pure NP solution under various strengths and driving frequencies of the RMF were also implemented as control. Figure 9 shows again that the thrombolytic effect of pure NPs without urokinase is very limited. It is noteworthy that a primary lysis speed of 13 μm/s was obtained, as shown in Figure 8b and Figure 9a,b. This is induced by the self-diffusion of a pure urokinase solution at a concentration of 30 μg/mL, and can be increased by increasing the concentration of urokinase, as shown in Figure 7. Furthermore, when the units of ω, H, and $C_{NP}$ are Hz, A/m, and mg/mL, respectively, the values of $bk\prime /D_{f}{}^{2}$ in Equation (2) can be obtained as $5.930\times 10^{-7}$, $5.928\times 10^{-7}$, and $5.935\times 10^{-7}$ by the fitting curves shown in Figure 8b and Figure 9a,b, respectively. Therefore, the value of $bk\prime /D_{f}{}^{2}$ can be regarded as $(5.931\pm 0.004)\times 10^{-7}$.

## 4. Conclusions

The introduction of magnetically controlled Fe_3_O_4_ NPs to promote the urokinase-administrated thrombolysis efficiency has been discussed in detail. The NPs are first aggregated into microrods under a static magnetic field; then, they are guided by a rotating magnetic field to induce a vortex, which strengthens the diffusion of urokinase toward the surface of the thrombus and results in the acceleration of thrombus removal. The thrombolysis speed is related to urokinase concentration, NP concentration, magnetic field strength, and the driving frequency of the rotating magnetic field. The experimental results validate the proposed model and prove that NPs can indeed improve thrombolytic efficiency. The thrombolysis speed can be enhanced to nearly two times higher than that of pure urokinase. It is expected that this method of accelerating thrombolysis may be applied in vivo in the future.

## References

[1] Van Es N., Coppens M., Schulman S., Middeldorp S., Buller H.R. (2014). Direct oral anticoagulants compared with vitamin K antagonists for acute venous thromboembolism: Evidence from phase 3 trials. Blood.

[2] Heit J.A., Spencer F.A., White R.H. (2016). The epidemiology of venous thromboembolism. J. Thromb. Thrombolys..

[3] Plicht B., Konorza T.F., Kahlert P., Al-Rashid F., Kaelsch H., Jánosi R.A., Buck T., Bachmann H.S., Siffert W., Heusch G. (2013). Risk factors for thrombus formation on the Amplatzer Cardiac Plug after left atrial appendage occlusion. JACC Cardiovasc. Interv..

[4] Empana J., Boulanger C.M., Tafflet M., Renard J.M., Leroyer A.S., Varenne O., Prugger C., Silvain J., Tedgui A., Cariou A. (2015). Microparticles and sudden cardiac death due to coronary occlusion. The TIDE (Thrombus and Inflammation in sudden DEath) study. Eur. Heart J. Acute Cardiovasc. Care.

[5] Pan S., Tsai T., Chen W., Shen C., Tsuei Y. (2015). An acute cerebral venous sinus thrombosis: Successful treatment by combining mechanical thrombolysis with continuous urokinase infusion. Clin. Neuroradiol..

[6] Butcher K., Shuaib A., Saver J., Donnan G., Davis S.M., Norrving B., Wong K.L., Abd-Allah F., Bhatia R., Khan A. (2013). Thrombolysis in the developing world: Is there a role for streptokinase?. Int. J. Stroke.

[7] Goel S.K., Goel I., Agarwal S. (2017). Effectiveness and comparison of reteplase versus streptokinase thrombolytic agents in the patients of acute myocardial infarction. Int. J. Med. Sci. Public Health.

[8] Chapman S.N., Mehndiratta P., Johansen M.C., McMurry T.L., Johnston K.C., Southerland A.M. (2014). Current perspectives on the use of intravenous recombinant tissue plasminogen activator (tPA) for treatment of acute ischemic stroke. Vasc. Health Risk Manag..

[9] Chen G., Liu Y., Xie Y., Li J., Liu H., Sun L., Peng Y., Liu F. (2013). High dose urokinase against massive pulmonary embolism in nephrotic syndrome. Blood Coagul. Fibrinolysis.

[10] De Martino R.R., Moran S.L. (2015). The role of thrombolytics in acute and chronic occlusion of the hand. Hand Clin..

[11] Imaizumi T., Inamura S., Kohama I., Yoshifuji K., Nomura T., Komatsu K. (2013). Antithrombotic drug uses and deep intracerebral hemorrhages in stroke patients with deep cerebral microbleeds. J. Stroke Cerebrovasc. Dis..

[12] Charidimou A., Nicoll J.A., McCarron M.O. (2015). Thrombolysis-related intracerebral hemorrhage and cerebral amyloid angiopathy: Accumulating evidence. Front. Neurol..

[13] Tsivgoulis G., Zand R., Katsanos A.H., Turc G., Nolte C.H., Jung S., Cordonnier C., Fiebach J.B., Scheitz J.F., Klinger-Gratz P.P. (2016). Risk of symptomatic intracerebral hemorrhage after intravenous thrombolysis in patients with acute ischemic stroke and high cerebral microbleed burden: A meta-analysis. JAMA Neurol..

[14] Suo D., Jin Z., Jiang X., Dayton P.A., Jing Y. (2017). Microbubble mediated dual-frequency high intensity focused ultrasound thrombolysis: An In vitro study. Appl. Phys. Lett..

[15] Bader K.B., Gruber M.J., Holland C.K. (2015). Shaken and stirred: Mechanisms of ultrasound-enhanced thrombolysis. Ultrasound Med. Biol..

[16] De Saint Victor M., Carugo D., Coussios C., Stride E.P. (2015). Ultrasound-enhanced thrombolysis: Mechanistic observations. J. Acoust. Soc. Am..

[17] Miller D.L., Smith N.B., Bailey M.R., Czarnota G.J., Hynynen K., Makin I.R.S., Bioeffects C.O.T.A. (2012). Overview of therapeutic ultrasound applications and safety considerations. J. Ultrasound Med..

[18] Chen J., Liu C., Hsu H., Wu T., Lu Y., Ma Y. (2016). Magnetically controlled release of recombinant tissue plasminogen activator from chitosan nanocomposites for targeted thrombolysis. J. Mater. Chem. B.

[19] Watermann A., Brieger J. (2017). Mesoporous Silica Nanoparticles as Drug Delivery Vehicles in Cancer. Nanomaterials.

[20] Hwang A.A., Lu J., Tamanoi F., Zink J.I. (2015). Functional nanovalves on protein-coated nanoparticles for in vitro and in vivo controlled drug delivery. Small.

[21] Patra S., Roy E., Karfa P., Kumar S., Madhuri R., Sharma P.K. (2015). Dual-responsive polymer coated superparamagnetic nanoparticle for targeted drug delivery and hyperthermia treatment. ACS Appl. Mater. Interfaces.

[22] Frank L.A., Contri R.V., Beck R.C., Pohlmann A.R., Guterres S.S. (2015). Improving drug biological effects by encapsulation into polymeric nanocapsules. Wiley Interdiscip. Rev. Nanomed. Nanobiotechnol..

[23] Chang M., Lin Y., Gabayno J.L., Li Q., Liu X. (2017). Thrombolysis based on magnetically-controlled surface-functionalized Fe_3_O_4_ nanoparticle. Bioengineered.

[24] Korin N., Kanapathipillai M., Matthews B.D., Crescente M., Brill A., Mammoto T., Ghosh K., Jurek S., Bencherif S.A., Bhatta D. (2012). Shear-Activated Nanotherapeutics for Drug Targeting to Obstructed Blood Vessels. Science.

[25] Lavik E., Ustin J. (2012). Leveraging Shear Stress to Bust Clots with Nanoparticles. Science.

[26] Madaan K., Kumar S., Poonia N., Lather V., Pandita D. (2014). Dendrimers in drug delivery and targeting: Drug-dendrimer interactions and toxicity issues. J. Pharm. Bioallied Sci..

[27] Dawidczyk C.M., Kim C., Park J.H., Russell L.M., Lee K.H., Pomper M.G., Searson P.C. (2014). State-of-the-art in design rules for drug delivery platforms: Lessons learned from FDA-approved nanomedicines. J. Control. Release.

[28] Gabayno J.L.F., Liu D., Chang M., Lin Y. (2015). Controlled manipulation of Fe_3_O_4_ nanoparticles in an oscillating magnetic field for fast ablation of microchannel occlusion. Nanoscale.

[29] Wu K.C. (2012). CMR of microvascular obstruction and hemorrhage in myocardial infarction. J. Cardiovasc. Magn. R..

[30] Kloner R.A. (2017). The importance of no-reflow/microvascular obstruction in the STEMI patient. Eur. Heart J..

[31] Serša I., Vidmar J., Grobelnik B., Mikac U., Tratar G., Blinc A. (2007). Modelling the effect of laminar axially directed blood flow on the dissolution of non-occlusive blood clots. Phys. Med. Biol..

[32] Rosenbluth M.N., Berk H.L., Doxas I., Horton W. (1987). Effective diffusion in laminar convective flows. Phys. Fluids.

[33] Biswal S.L., Gast A.P. (2004). Micromixing with linked chains of paramagnetic particles. Anal. Chem..

[34] Chang M., Gabayno J.L.F., Ye R., Huang K., Chang Y. (2017). Mixing efficiency enhancing in micromixer by controlled magnetic stirring of Fe_3_O_4_ nanomaterial. Microsyst. Technol..

